# WNK1-OSR1 kinase-mediated phospho-activation of Na^+^-K^+^-2Cl^-^ cotransporter facilitates glioma migration

**DOI:** 10.1186/1476-4598-13-31

**Published:** 2014-02-20

**Authors:** Wen Zhu, Gulnaz Begum, Kelli Pointer, Paul A Clark, Sung-Sen Yang, Shih-Hua Lin, Kristopher T Kahle, John S Kuo, Dandan Sun

**Affiliations:** 1 Department of Neurology, University of Pittsburgh, Pittsburgh, PA, USA; 2 Cellular and Molecular Biology Program, University of Wisconsin School of Medicine and Public Health, Madison, WI, USA; 3 Department of Neurological Surgery, University of Wisconsin School of Medicine and Public Health, Madison, WI, USA; 4 Carbone Cancer Center, University of Wisconsin, Madison, WI, USA; 5 Division of Nephrology, Department of Medicine, Tri-Service General Hospital, National Defense Medical Center, Taipei, Taiwan; 6 Department of Neurological Surgery, Massachusetts General Hospital and Harvard Medical School, Boston, MA, USA; 7 Veterans Affairs Pittsburgh Health Care System, Geriatric Research, Educational and Clinical Center, Pittsburgh, PA, USA; 8 Department of Neurology, University of Pittsburgh, S-598 South Biomedical Science Tower (BST), 3500 Terrace Street, Pittsburgh, PA 15213, USA

**Keywords:** Bumetanide, Cell volume, Ezrin, Ion cotransporter, Temozolomide

## Abstract

**Background:**

The bumetanide (BMT)-sensitive Na^+^-K^+^-2Cl^-^ cotransporter isoform 1 (NKCC1) maintains cell volume homeostasis by increasing intracellular K^+^ and Cl^-^ content via regulatory volume increase (RVI). Expression levels of NKCC1 positively correlate with the histological grade and severity of gliomas, the most common primary adult brain tumors, and up-regulated NKCC1 activity facilitates glioma cell migration and apoptotic resistance to the chemotherapeutic drug temozolomide (TMZ). However, the cellular mechanisms underlying NKCC1 functional up-regulation in glioma and in response to TMZ administration remain unknown.

**Methods:**

Expression of NKCC1 and its upstream kinases With-No-K (Lysine) kinase 1 (WNK1) and oxidative stress-responsive kinase-1 (OSR1) in different human glioma cell lines and glioma specimens were detected by western blotting and immunostaining. Live cell imaging and microchemotaxis assay were applied to record glioma cell movements under different treatment conditions. Fluorescence indicators were utilized to measure cell volume, intracellular K^+^ and Cl^-^ content to reflect the activity of NKCC1 on ion transportation. Small interfering RNA (siRNA)-mediated knockdown of WNK1 or OSR1 was used to explore their roles in regulation of NKCC1 activity in glioma cells. Results of different treatment groups were compared by one-way ANOVA using the Bonferroni post-hoc test in the case of multiple comparisons.

**Results:**

We show that compared to human neural stem cells and astrocytes, human glioma cells exhibit robust increases in the activation and phosphorylation of NKCC1 and its two upstream regulatory kinases, WNK1 and OSR1. siRNA-mediated knockdown of WNK1 or OSR1 reduces intracellular K^+^ and Cl^-^ content and RVI in glioma cells by abolishing NKCC1 regulatory phospho-activation. Unexpectedly, TMZ activates the WNK1/OSR1/NKCC1 signaling pathway and enhances glioma migration. Pharmacological inhibition of NKCC1 with its potent inhibitor BMT or siRNA knockdown of WNK1 or OSR1 significantly decreases glioma cell migration after TMZ treatment.

**Conclusion:**

Together, our data show a novel role for the WNK1/OSR1/NKCC1 pathway in basal and TMZ-induced glioma migration, and suggest that glioma treatment with TMZ might be improved by drugs that inhibit elements of the WNK1/OSR1/NKCC1 signaling pathway.

## Background

Glioblastoma multiforme (GBM) is the most common malignant primary brain tumor in adults. The standard treatment of malignant glioma includes maximal surgical resection followed by concurrent radiation and chemotherapy with temozolomide (TMZ) [1]. Despite aggressive treatment, GBM patients have a poor median survival of 14 months [2]. The highly infiltrative behavior of gliomas causes difficulties in achieving complete surgical resections. Recurrence of the disease is attributed in part to resistance of glioma cells to the standard chemotherapeutic reagent TMZ [3]. It is important to identify new therapeutic targets to hinder the migration of the invasive glioma cells and sensitize glioma cells to chemotherapy.

Ion channels and ion transporters have emerged to play an important role in tumorigenesis, glioma migration and metastasis [4]. Expression of Na^+^-K^+^-2Cl^-^ cotransporter isoform 1 (NKCC1) in human glioma has been shown to positively correlate with the tumor grades. NKCC1 is involved in glioma migration through regulation of focal adhesion dynamics, cell contractility, and cell volume [5-7]. Pharmacological inhibition or shRNA-mediated knockdown of NKCC1 decreases glioma cell migration and invasion [5,7]. Recently, we reported that NKCC1 activity is important in GC survival [8]. NKCC1 is the key ion transporter in regulation of intracellular K^+^ (K^+^_i_), Cl^-^ (Cl^-^_i_) and cell volume in primary glioma cells (GCs) and glioma stem cells (GSCs) [8]. Most importantly, TMZ stimulates NKCC1 activity to counteract loss of K^+^_i_ and Cl^-^_i_ and apoptotic volume decrease (AVD) during early apoptosis [8]. Inhibition of NKCC1 activity with its potent inhibitor bumetanide (BMT) enhanced TMZ-mediated apoptosis in both GCs and GSCs [8]. However, the mechanisms underlying NKCC1 up-regulation in glioma, and how NKCC1 activity is modulated by TMZ, are unknown.

Activation of NKCC1 protein is regulated by a family of kinases named the With-No-K (Lysine) kinases (WNKs, WNK1-4) [9]. To date, the best characterized substrates of WNKs include two mammalian protein kinases in the germinal center kinase-VI subfamily, SPS1-related proline/alanine-rich kinase (SPAK) and oxidative stress-responsive kinase 1 (OSR1) [9]. In our previous study, we documented that TMZ treatment triggered increased phosphorylation of WNK1 in both GCs and GSCs [8]. But, it has not yet been defined whether SPAK and/or OSR1 are the intermediate regulatory kinases in modulating NKCC1 function in GCs.

In the present study, we investigated whether WNK1-SPAK/OSR1 signaling pathway regulates NKCC1 activity in GCs and whether this signaling pathway is involved in regulation of glioma migration, with and without chemotherapeutic treatment. We report here that WNK1 and OSR1 are the dominant upstream regulatory kinases of NKCC1 in glioma cells. Moreover, the WNK1/OSR1/NKCC1 signaling pathway plays an important role in glioma migration and is stimulated by TMZ. These findings illustrate significant potentials of this signal transduction pathway as new therapeutic targets for combined chemoradiotherapy for GBM.

## Results

### Abundant expression of WNK1/OSR1/NKCC1 proteins in glioma cells

**Figure 1:**
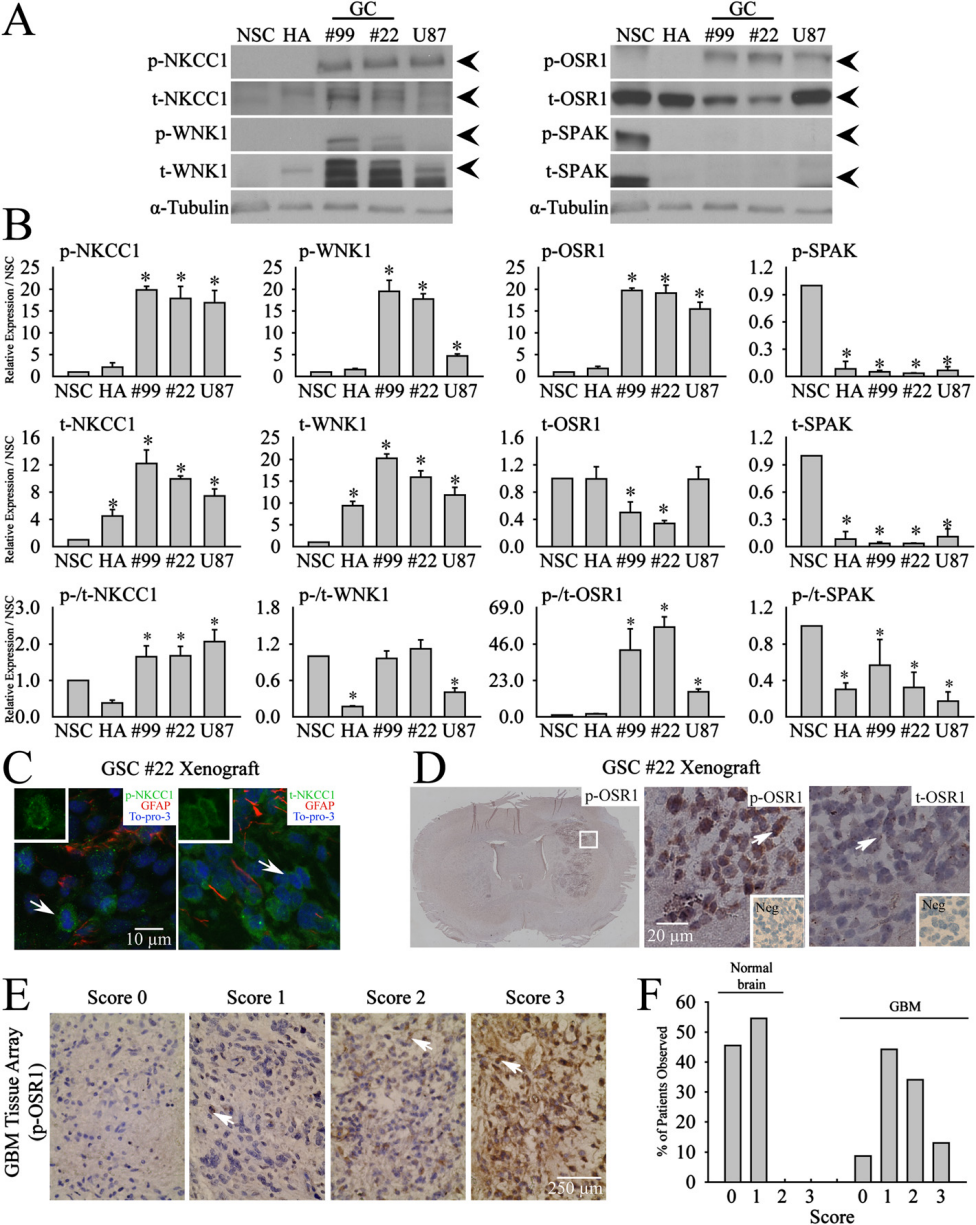
**Abundant expression of WNK1/OSR1/NKCC1 in primary glioma cells and GBM tissues. A**. Representative immunoblots for expression of either phosphorylated (p-) or total (t-) NKCC1, WNK1, OSR1 and SPAK in human neural stem cell (NSC), human astrocyte (HA), primary GC#99, GC#22 and U87. **B**. Summary data of immunoblotting. Upper and middle panels, expression of each protein was first normalized by α-tubulin and relative expression level in each cell type was then normalized to NSC. Lower panel, expression of each phosphorylated protein was first normalized by its total protein and relative expression level in each cell type was then normalized to NSC. Data are mean ± SEM. n = 3, *p < 0.05 vs. NSC. **C**. Representative immunofluorescent staining of p-NKCC1, t-NKCC1 in xenograft brain tissues of SCID mouse derived from glioma stem cell (GSC#22). Arrow, representative cells with positive staining of protein of interest. Insets: images with higher magnification. **D**. Representative immunostaining of p-OSR1, t-OSR1 in xenograft brain tissues of SCID mouse derived from glioma stem cell (GSC#22). White box in whole-brain images indicates the corresponding location of acquisition for high magnification photomicrographs. Arrow, representative cells with positive staining of protein of interest. Insets: negative controls with primary antibodies omitted. **E**. Representative images of p-OSR1 immunohistochemistry with different p-OSR1 immunohistochemistry staining intensity in a tissue microarray (TMA) of GBM. Arrow, representative cells with positive staining of protein of interest. **F**. Summary data of percentage of patients observed with different p-OSR1 expression scores with either normal or GBM group.

First, we characterized expression of WNK1, SPAK, OSR1, and NKCC1 protein in human neural stem cells (NSC), human astrocytes (HA), primary glioma cells (GC#99 and GC#22), and GBM cell line U87. As shown in Figure 1A and B, NSC and HA showed relative low expression of p-NKCC1 and t-NKCC1. In contrast, all three glioma cell lines exhibited abundant expression of both proteins. Normalized by the expression level in NSC, p-NKCC1 protein was 17.6 ± 3.1 folds higher in U87, 20.1 ± 1.2 folds higher in GC#99, and 18.5 ± 1.7 folds in GC#22. The expression of t-NKCC1 ranged from 7.9 ± 1.0 folds in U87 to 12.1 ± 2.7 folds in GC#99. Similar abundant expression of p-WNK1 and t-WNK1 was also detected in GCs. p-WNK1 was 4 ~ 20 folds more abundant in GCs than in NSC and t-WNK1 was 12.5 ~ 20 folds higher in GCs (Figure 1A and B). In contrast, NSC expressed relatively higher level of t-OSR1. GC#99 only contained 47.6 ± 9% of t-OSR1 (p < 0.05) and GC#22 had 31.4 ± 2% of t-OSR1, compared to NSC (p < 0.05). Interestingly, the basal expression of p-OSR1 remained high in both primary glioma cell lines as well as in U87 (p < 0.05) (Figure 1A and B). Moreover, expression of p-SPAK and t-SPAK was barely detectable in all three glioma cell lines and in HA (Figure 1A and B). The presence of trace p-SPAK and t-SPAK signals in GC#99, GC#22 and U87 samples was revealed when ECL exposure time was increased to 3 h (Additional file 1: Figure S1).

Expression of NKCC1 and OSR1 protein was also detected in GBM xenograft tissues in SCID mouse brains derived from human GSC#22. As shown in Figure 1C, almost all cells within the human GBM xenografts exhibited positive immunostaining for p-NKCC1, and t-NKCC1 (Figure 1C). Moreover, p-OSR1 was abundantly expressed in GBM xenograft tissues or GBM tissue array samples (Figure 1D and E). Normal brain samples exhibited no or low level of p-OSR1 immunoreactive signals. In contrast, ~50% of GBM biopsies showed moderate to strong p-OSR1 expression (Figure 1F). Taken together, we concluded that GCs express abundant p-WNK1, p-OSR1 and p-NKCC1 proteins, but not SPAK protein. In the rest of our study, we investigated regulation and function of the WNK1/OSR1/NKCC1 signaling cascade in GCs.

### NKCC1 activity in GC migration in the absence and presence of TMZ treatment

**Figure 2:**
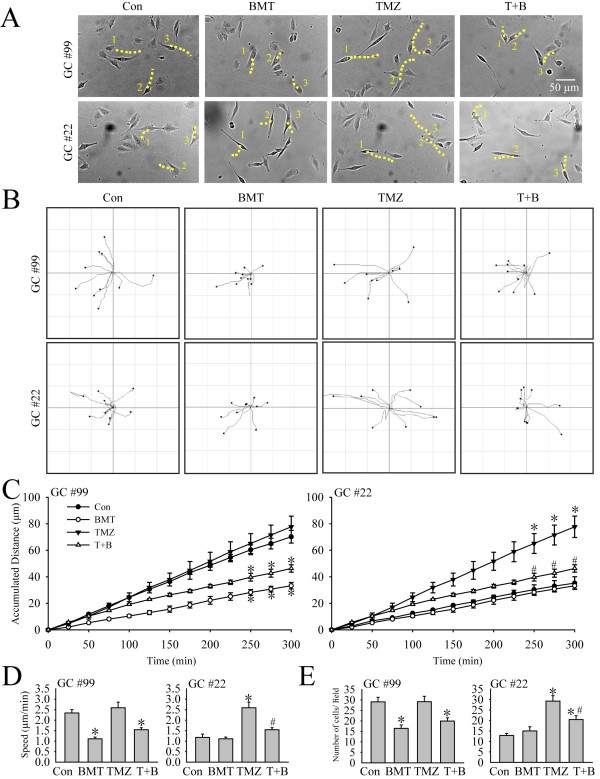
**Inhibition of NKCC1 activity abolishes glioma motility and serum-induced microchemotaxis in response to TMZ. A**. Representative images of cell random movment in the presence of control medium (DMEM + 10 % FBS) (Con), 10 μM BMT (BMT), 100 μM TMZ (TMZ), or 100 μM TMZ plus 10 μM BMT (T + B) for 5 h. Three moving cells in the field were marked (#1-3). Yellow dashed line: changes of traces of cell gravity center with in 5 h. **B**. Motility of glioma cells was recorded using the Nikon TiE time-lapse imaging system. Random moving traces of 10 representative cells were shown under Con, BMT, TMZ, or T + B conditions in the 300-min recording period. **C**. Summary data of GC motility. Accumulated distance of GC cell movement during 0–300 min was calculated in each condition. Data were mean ± SEM (n =4). *p < 0.05 vs. Con. #p < 0.05 vs. TMZ. **D**. Summary data of average speed of GC movement in 5 h. Data were mean ± SEM (n =4). *p < 0.05 vs. Con. #p < 0.05 vs. TMZ. **E**. Serum-induced microchemotaxis of GC#99 and GC#22 was determined using the Boyden Chamber (8 μm pore) for 5 h under different treatment conditions (Con, BMT, TMZ, or TMZ plus BMT). Summary data of numbers of migrated cells per field in different treatment groups. Data are mean ± SEM. n = 6, *p < 0.05 vs. Con. #p < 0.05 vs. TMZ.

Random cell movements were recorded with time-lapse imaging technique. In the current study, TMZ at a concentration of 100 μM was chosen because it is similar to the serum level of ~ 100 μM during clinical TMZ treatment [10] and has been characterized in our previous study [8]. Figure 2A illustrated individual glioma cell moving traces in 5 h under different conditions (Con, 10 μM BMT, 100 μM TMZ, or 100 μM TMZ plus 10 μM BMT). Many cells displayed position changes during the 5-h period (dashed lines). Figure 2B further illustrates the random moving traces of GCs, showing that the motility of GC#99 was clearly inhibited when NKCC1 activity was blocked with BMT under either control conditions or in the presence of TMZ. Moreover, the motility of GC#22 appeared to be increased in the presence of TMZ, but, this stimulation was attenuated by inhibiting NKCC1 with BMT treatment. The summarized data in Figure 2C illustrated that BMT significantly reduced the basal level of GC#99 mobility by 56% under control conditions (70.2 ± 4.8 μm vs 33.3 ± 2.4 μm travel distance, p < 0.05). Moreover, BMT also suppressed the GC#99 motility under TMZ-treated conditions (46.3 ± 2.8 μm, p < 0.05) (Figure 2D). On the other hand, GC#22 exhibited a low basal motility under control conditions (travel distance of 35.23 ± 4.80 μm in 5 h). BMT treatment had no effects on the basal motility (neither on the total travel distance nor the speed, Figure 2C and D). Interestingly, in the presence of TMZ, GC#22 cell mobility was increased by 216 ± 9.1% of control (77.7 ± 8.0 μm in 5 h, p < 0.05). The mobility rate was doubled from 1.17 to 2.59 μm/min. Most importantly, inhibition of NKCC1 activity with BMT abolished this stimulation in GC#22 (p < 0.05, Figure 2C and D).

To further validate these phenomena, we examined migration behaviors of GC#99 and GC#22 in the serum-induced microchemotaxis assay. Consistent with their motility profiles with live cell imaging, GC#99 exhibited a higher basal cell migration level (29.1 ± 2.1 cells/field, Figure 2E). BMT decreased GC#99 migration by 56.3 ± 7.4% (p < 0.05). TMZ treatment did not change the migration rate of GC#99, but BMT remains effective in reducing GC#99 migration in the presence of TMZ (Figure 2E, left panel). In contrast, GC#22 exhibited lower basal migratory ability through the 8-μm transwell membrane under control conditions (12.8 ± 1.8 cells/field, Figure 2E, right panel). Inhibition of NKCC1 had no effects on the basal level of GC#22 migration. However, the number of migrated cells of GC#22 significantly increased in the presence of TMZ (29.2 ± 2.5 cells/field, p < 0.05). Inhibition of NKCC1 with BMT treatment significantly attenuated the TMZ-mediated stimulation of GC#22 migration (p < 0.05, Figure 2E, right panel). The commercial GBM cell line U87 exhibited similar migratory pattern as GC#22 (Additional file 1: Figure S2). Taken together, these studies revealed that GC#99 and GC#22 exhibited heterogeneity in basal mobility, migration and sensitivity to NKCC1 inhibition and TMZ treatments. These findings led us to further investigate how NKCC1 protein is regulated in GC#99 and GC#22 in response to TMZ treatment.

### TMZ stimulates the WNK1/OSR1/NKCC1 signal transduction pathway in GCs

**Figure 3:**
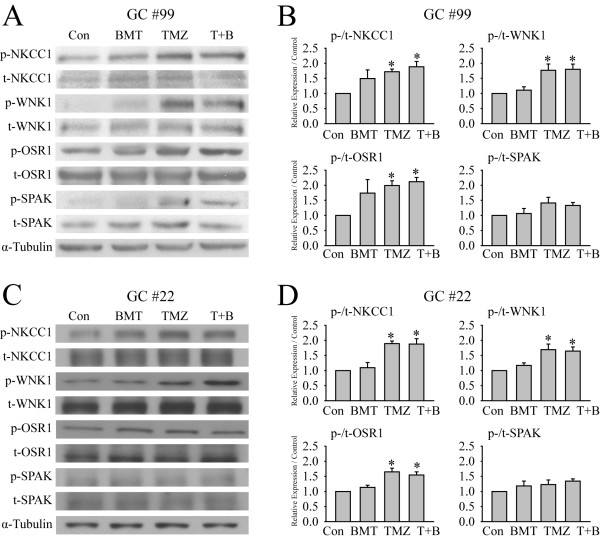
**TMZ treatment activates the WNK1/OSR1/NKCC1 signaling pathway in GC#99 and GC#22. A** and **C**. Representative immunoblots showing increased expression of p-WNK1, p-OSR1 and p-NKCC1 proteins in response to TMZ exposure in GC#99 and GC#22. GCs were exposed to control medium (Con), 10 μM bumetanide (BMT), 100 μM TMZ, or 100 μM TMZ plus 10 μM BMT (T + B) for 4 h. **B** and **D**. Summary data of immunoblotting. Expression of each phosphorylated protein was first normalized by its total protein, and relative expression levels with different treatments were then normalized to Con. Data are mean ± SEM. n = 4–5, *p < 0.05 vs. Con.

In order to understand how NKCC1 protein is regulated in GC#99 and GC#22 in response to TMZ, we first examined whether TMZ stimulates the WNK1/OSR1 signaling pathway in GCs. As shown in Figure 3A, exposing GC#99 to TMZ for 4 h triggered an increase of p-NKCC1 expression and a concurrent change of the upstream kinases p-WNK1 and p-OSR1. Figure 3B shows that p-WNK1 was increased by 176.7 ± 20.6% of control (p < 0.05), p-OSR1 by 199.2 ± 15.7% of control (p < 0.05), and p-NKCC1 by 171.9 ± 8.9% of control (p < 0.05) after TMZ treatment. However, p-SPAK and the total protein level of each tested protein in GC#99 were not significantly altered by TMZ (Figure 3A, B and Additional file 1: Figure S3). Moreover, the combined treatment of TMZ and BMT did not affect the TMZ-induced up-regulation of p-WNK1, p-OSR1 or p-NKCC1 in GC#99.

In the case of GC#22, TMZ triggered similar activation patterns of the WNK1/OSR1/NKCC1 cascade. The p-WNK1 expression was increased by 169.1 ± 18.6% of control (p < 0.05) and p-OSR1 was elevated by 170.0 ± 12.4% of control (p < 0.05) and p-NKCC1 was by 189.4 ± 8.4% of control (p < 0.05, Figure 3C and D). Moreover, t-WNK1, t-OSR1, t-NKCC1 and t-SPAK remained unchanged in both TMZ-treated and TMZ + BMT-treated cells (Additional file 1: Figure S3). Last, BMT treatment did not affect the TMZ-mediated elevation of p-WNK1, p-OSR1 or p-NKCC1 in GC#22. No changes of p-SPAK were observed in the TMZ-treated GC#22. In summary, TMZ triggered activation of the WNK1/OSR1/NKCC1 signaling pathway in both GC#99 and GC#22, while SPAK protein was not activated and likely plays a minimal role in these cells.

### Down-regulation of the WNK1/OSR1 pathway abolishes the TMZ-induced NKCC1 activation

**Figure 4:**
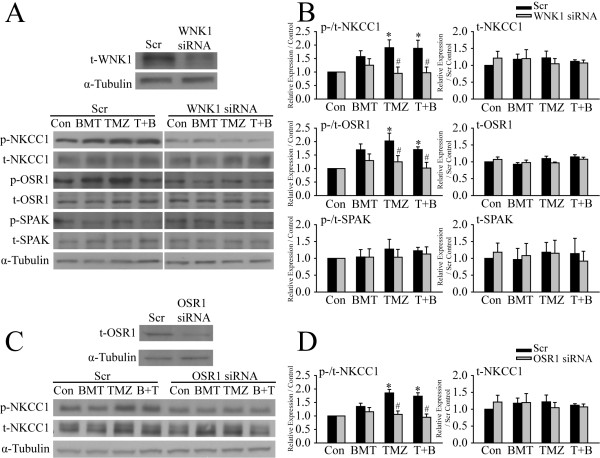
**siRNA-mediated down-regulation of WNK1/OSR1 attenuates the TMZ-induced NKCC1 activation. A**. Representative immunoblots showing effects of WNK1 siRNA treatment on expression of t-WNK1 (upper panel) and p-NKCC1, p-OSR1 and p-SPAK (lower panel). **C**. Representative immunoblots showing effects of OSR1 siRNA treatment on expression of t-OSR1 (upper panel) and p-NKCC1 (lower panel). GC#99 were treated with either scramble siRNA (Scr), WNK1 siRNA, or OSR1 siRNA for 48 h, followed by exposure to control medium (Con), 10 μM BMT, 100 μM TMZ, or 100 μM TMZ plus 10 μM BMT (T + B) for 4 h. **B** and **D**. Summary data of immunoblotting. Left panel, the expression level of each phosphorylated protein was normalized by its total protein and then the basal expression under control conditions. Right panel, the expression level of each total protein was normalized by α-tubulin and then the basal expression in Scr-treated cells under control conditions. Data are mean ± SEM, n = 3 ~ 5, *p < 0.05 vs. Con, #p < 0.05 vs. Scr.

To further determine that WNK1 and OSR1 are the upstream kinases regulating NKCC1 activity in GCs, siRNA knockdown approach was used to selectively reduce protein expression of either WNK1 or OSR1 in GC#99 cells. Compared to scramble siRNA (Scr)-treated cells, expression of t-WNK1 in the WNK1 siRNA-treated cells was reduced by ~ 50% (48.3 ± 5.3% scr, p < 0.05, Figure 4A). WNK1 siRNA treatment did not alter the expression levels of t-NKCC1, t-OSR1 and t-SPAK. As expected, down-regulation of WNK1 in GC#99 lowered the expression of p-NKCC1 across all four conditions (Con, BMT, TMZ or T + B, Figure 4A). Most importantly, TMZ failed to induce elevation of p-NKCC1 expression in the WNK1 siRNA-treated GC#99 (95.2 ± 23.1% of Con WNK1 siRNA-treated cells, Figure 4A and B). Moreover, down-regulation of WNK1 in GC#99 also significantly attenuated the TMZ-induced activation of OSR1 (124.8 ± 28.1% in WNK1 siRNA-treated cells vs. 202.4 ± 28.9% in the Scr-treated cells) (Figure 4A and B). Expression of p-SPAK was not significantly changed in either Scr-siRNA or WNK1 siRNA-treated cell. Taken together, these findings suggest that WNK1 is the major WNK isoform regulating NKCC1 in GC#99 and that WNK1 activation is required for the TMZ-mediated NKCC1 stimulation.

We then determined whether OSR1 is the intermediate player between WNK1 and NKCC1. After 48 h of OSR1 siRNA treatment, t-OSR1 expression was reduced by ~ 60% (57.2 ± 7.1% of Scr, p < 0.05), while t-NKCC1 expression was not affected (Figure 4C and D). TMZ failed to stimulate p-NKCC1 expression in the OSR1 siRNA-treated GC#99 (p > 0.05, Figure 4C and D). These data further suggest that OSR1 is downstream of WNK1 and collectively regulates NKCC1 activity in GCs.

### Down-regulation of WNK1/OSR1 reduces microchemotaxis of GCs

**Figure 5:**
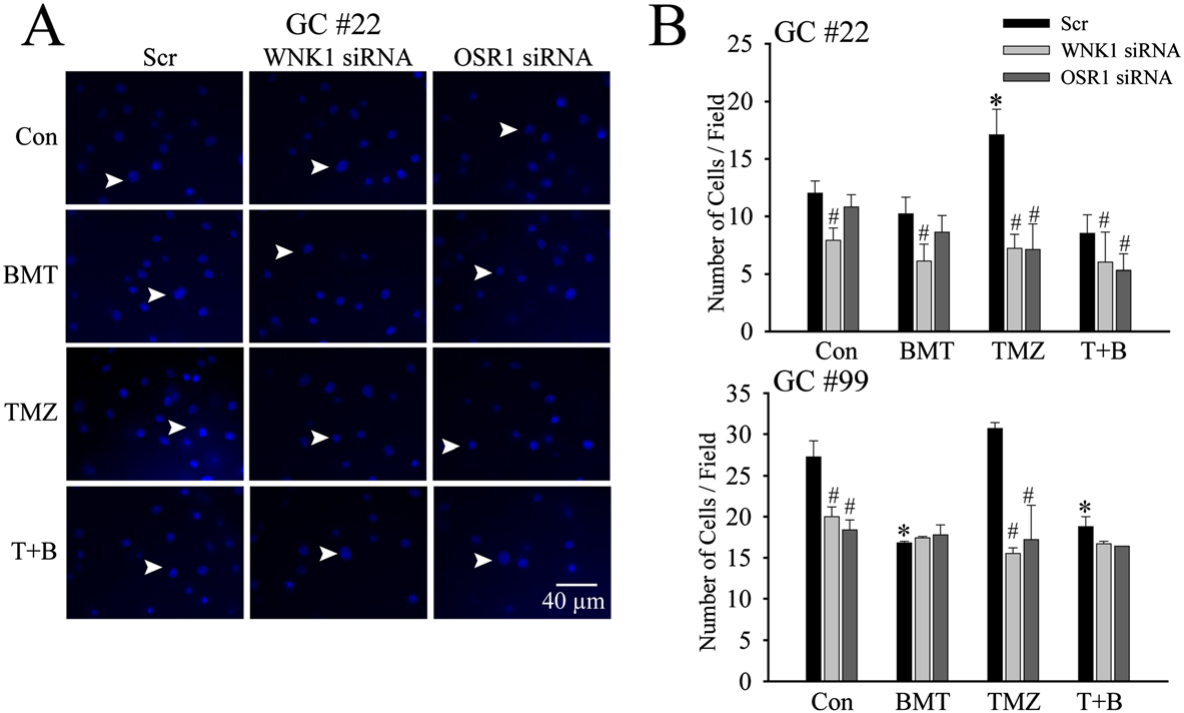
**siRNA-mediated down-regulation of WNK1/OSR1 reduces microchemotaxis of GCs. A**. After 48 h treatment with either scramble siRNA (Scr), WNK1 siRNA, or OSR1 siRNA, serum-induced microchemotaxis of GC#22 was determined for 5 h under different treatment conditions control medium (Con), 10 μM BMT, 100 μM TMZ, or 100 μM TMZ plus 10 μM BMT (T + B). Representative images of GC#22 that have migrated through an 8-μm transwell barrier after 5 h were shown. **B**. Summary data of numbers of migrated GC#22 and GC#99 cells in different treatment groups. Data are mean ± SEM. n = 4, *p < 0.05 vs. Con. #p < 0.05 vs. Scr.

Given the important role of WNK1 and OSR1 in regulation of NKCC1 in GCs, we further investigated whether reduced expression of these upstream kinases will affect the migratory behaviors of GCs, especially under TMZ treatment. Figure 5A illustrated the representative images of GC#22 that migrated through the membrane in the serum-induced microchemotaxis assay. Compared to Scr-treated cells, the number of migrated cells was clearly less in the WNK1 siRNA-treated cells as well as in the OSR1 siRNA-treated cells. Figure 5B is the summarized data of the transwell-migration of both GC#22 and GC#99. In GC#22, reduced expression of WNK1 not only inhibited the TMZ-induced increase in migration (7.2 ± 1.2 cells/field vs. 17.1 ± 2.2 cells/field of Scr-treated cells, p < 0.05), but also led to significant reduction in cell migration across other three conditions (Con, BMT and T + B) (p < 0.05, Figure 5B, upper panel). Similarly, TMZ failed to stimulate cell migration in the OSR1-knockdown GC#22 (p < 0.05, Figure 5B, upper panel). GC#99 exhibited similar results that knockdown of either WNK1 or OSR1 protein expression significantly decreased cell migration under both Con and TMZ conditions (p < 0.05, Figure 5B, lower panel). But, inhibition of NKCC1 with BMT had no further effects on reducing GC#99 migration. Taken together, these data strongly suggest that WNK1/OSR1/NKCC1 signaling pathway plays an important role in regulation of basal motility in GCs, and TMZ stimulates this signaling pathway and promotes GC migration.

### Down-regulation of WNK1 reduced [K^+^]_i_, [Cl^-^]_i_, and impaired cell volume regulation in GCs

**Figure 6:**
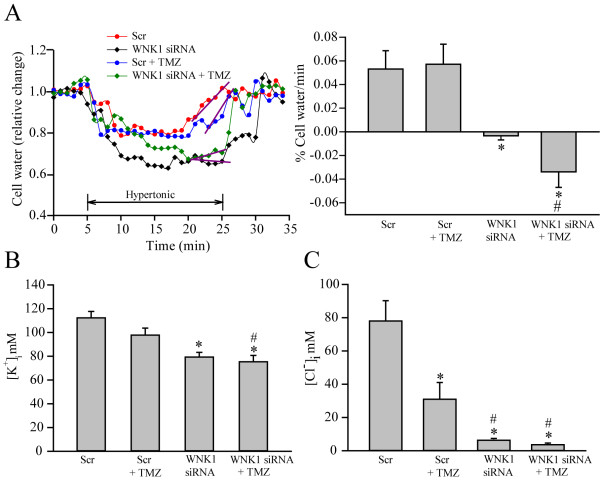
**Inhibition of WNK1 abolishes NKCC1-mediated regulatory volume increase and K**^**+**^_**i **_**and Cl**^**-**^_**i **_**homeostasis. A**. Cell water content and relative cell volume were determined using the fluorescent dye calcein as described in Methods. GC#99 was treated with control medium, scramble siRNA (Scr) or WNK1 siRNA for 48 h. To induce RVI, cells were exposed to isotonic HEPES-MEM (310 mOsm, 5 min), hypertonic HEPES-MEM (370 mOsm, 25 min), and isotonic HEPES-MEM (5 min). The slope of the RVI was determined by fitting a regression line (in purple) to the cell volume recovery at ~ 20–25 min after exposure to hypertonic stress. Right panel, summary data of RVI. Data are means ± SEM. n = 3–4. **B**. Effects of WNK1 siRNA on [K^+^]_i_ in GC#99. [K^+^]_i_ was determined using the fluorescent probe PBFI. Data are means ± SEM, n = 3. *p <0.05 vs. Scr. **C**. Effects of WNK1 siRNA on [Cl^-^]_i_ in GC#99. [Cl^-^]_i_ was determined using the fluorescent probe MQAE. Data are means ± SEM, n = 4. *p < 0.05 vs. Con; #p < 0.05 vs. Scr + TMZ.

We speculated that the WNK1/OSR1/NKCC1 signaling pathway functions in regulation of GC migration via changing K^+^_i_, and Cl^-^_i_ ionic homeostasis and cell volume. We examined whether knockdown of the WNK1 affects GC ionic contents and cell volume regulation. As shown in Figure 6A, exposing Scr-treated GC#99 cells to hypertonic HEPES-MEM (367 mOsm) induced ~ 30 ± 4% cell shrinkage in 3–5 min. This initial cell shrinkage is mediated by osmotically obligated H_2_O loss. Then, cells started to recover cell volume by the process of RVI. Cell volume started recovering at ~ 10–12 min at a rate of 0.05 ± 0.01% vol/min and nearly completely recovered by 25 min (a classical RVI response). In contrast, RVI response was completely abolished in the WNK1 siRNA-treated cells either in the absence or presence of TMZ (Figure 6A, purple slope). We have previously shown that RVI in glioma is largely mediated by the activity of NKCC1 [8]. Therefore, in the WNK1 siRNA-treated cells, NKCC1 activity was compromised and the cells failed to recover the volume. The shrinkage response in the WNK1 siRNA-treated cells appeared to be more profound, which may be due to the additional activation of outwardly directed K^+^-Cl^-^ cotransporter and loss of K^+^ and Cl^-^ in response to down-regulation of WNK1, as the WNK1-mediated phosphorylation of K^+^-Cl^-^ cotransporter inhibits its function [11].

Moreover, down-regulation of WNK1 also led to ~30 ± 2.9% loss of basal [K^+^]_i_ (Figure 6B). TMZ did not cause additional reduction in [K^+^]_i_ ( ~35% loss of [K^+^]_i_ of controls, Figure 6B). In contrast, TMZ treatment decreased [Cl^-^]_i_ by ~ 61% GC. Down regulation of WNK1 by siRNA alone or combined with TMZ treatment further lowered [Cl^-^]_i_ by ~ 92% (Figure 6C). These results clearly suggest that the upstream WNK1 kinase plays an essential role in maintaining [K^+^]_i_ and [Cl^-^]_i_ as well as cell volume regulation of GCs. Therefore, these functions could involve in GC migration.

### NKCC1 phosphorylation and its interaction with ezrin, radixin, and moesin protein complexes

**Figure 7:**
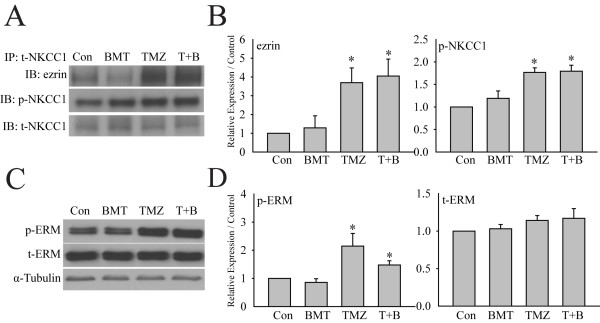
**Interactions between NKCC1 and p-ERM in GCs. A**. Representative immunoblots showing immunoprecipitation of ezrin, p-NKCC1, or t-NKCC1 proteins in GCs. GC#22 cells were exposed to control medium (Con), 10 μM bumetanide (BMT), 100 μM TMZ, or 100 μM TMZ plus 10 μM BMT (T + B) for 4 h. **B**. Summary data of immunoblotting. Expression of each protein was first normalized by t-NKCC1. Relative expression level under different treatments was normalized to Con. Data are mean ± SEM. n = 4, *p < 0.05 vs. Con. **C**. Representative immunoblots showing expression of p-ERM and t-ERM proteins in GC#22. **D**. Summary data of immunoblotting. Expression of each protein was first normalized by α-tubulin. Relative expression level under different treatments was then normalized to Con. Data are mean ± SEM. n = 5, *p < 0.05 vs. Con.

ERM (Ezrin, Radixin, and Moesin) proteins play an important role in cancer migration and invasion and interact with NKCC1 protein in glioma migration [5,12]. We speculate that WNK1/OSR1-mediated phosphorylation of NKCC1 protein may alter its interaction with ERM complex and promote glioma cell migration. As shown in Figure 7A, in GC#22 cells, p-NKCC1 protein in the immunoprecipitation fractions was significantly increased in TMZ- or TMZ + BMT-treated group (Figure 7A). Interestingly, ~ 4 fold increase in ezrin level was also detected in the immunoprecipitation fractions of TMZ- or TMZ + BMT-treated cells (p < 0.05, Figure 7A and B). Moreover, p-ERM but not t-ERM was significantly increased in GCs treated with TMZ or TMZ plus BMT (Figure 7B, p < 0.05). Taken together, these findings suggest that there is an increased interaction between p-NKCC1 and ezrin in GCs, which may promote glioma cell migration in the presence of TMZ. The phosphorylation of both NKCC1 and ERM proteins may facilitate their interactions.

## Discussion

### Increased phosphorylation of WNK1 and OSR1 in glioma cells

WNK1-SPAK/OSR1 signaling pathway is evolutionarily conserved regulators of ion transport and cell volume by altering the net phosphorylation state of ion transporters [13]. Moreover, WNK1 has been identified as an important kinase involved in development and cancer [14-16]. Mice with homozygous Wnk1 mutation died during embryonic development [15]. In Hela cells, WNK1 is required for mitosis and abscission [14]. Depletion of WNK1 with siRNA led to aberrant mitotic spindles, defective abscission and reduced cell survival [14]. WNK1 kinase expression is also found to correlate with invasiveness in F11 neural tumor cells [16]. A dramatic decrease of WNK1 expression was observed in the cells with a reduced rate of cell migration and invasion [16].

In the current study, compared to NSC and HA, we detected increased expression of p-WNK1, t-WNK1 and p-OSR1 protein in the GBM cell lines. Abundant expression of p-OSR1 and p-NKCC1 was also revealed in GBM xenografts and GBM tissue microarray samples. But, expression of SPAK protein was barely detectable in GCs, which are consistent with the reports in Hela cells or other glioma cell lines and glioma specimens [6,17]. p-WNK1 and t-WNK1 expression was not examined in GBM xenografts or GBM tissue arrays in this study because no commercial antibodies of WNK1 are specific for immunostaining. Taken together, these findings suggest that the WNK1/OSR1/NKCC1 signal pathway may be important in pathogenesis of glioma.

### WNK1 and OSR1 are the dominant upstream kinases in regulating NKCC1 in glioma cells

NKCC1 activity is controlled by protein phosphorylation and dephosphorylation [11]. WNK1/SPAK/OSR1 signaling pathway is the well-studied upstream regulatory component of NKCC1 [13]. WNK1 is a serine/threonine protein kinase, which is activated upon hypertonic stress, low [Cl^-^]_i_ or isotonic cell shrinkage, and plays an important role in regulation of SLC12 gene family including NKCC [18]. On the other hand, SPAK and OSR1 are two well-characterized WNK1 substrates [9]. In response to osmotic stress, WNK1 interacts with SPAK/OSR1 and phosphorylates them in two sites (Thr^233^ and Ser^373^ in human SPAK, Thr^185^ and Ser^325^ in human OSR1 [19]. The phosphorylation triggers activation of SPAK and OSR1, which in turn stimulates NKCC1 to maintain intracellular ionic strength and volume [9].

In the current study, we found that knockdown of WNK1 or OSR1 with siRNA significantly reduced basal levels of p-NKCC1 protein in GCs. Reduction of either WNK1 or OSR1 via siRNAs abolished the TMZ-induced phosphorylation and activation of NKCC1 in GCs. These data suggest that the WNK1 plays a dominant role in regulating NKCC1 protein in GCs. We also concluded that OSR1 is the major intermediate player between WNK1 and NKCC1 because of the low level of SPAK in GCs. Furthermore, TMZ treatment at a relatively low dose is sufficient to stimulate the activities of these two kinases in glioma cells. Interestingly, among seven recurrent GBMs in our TMA analysis, all six biopsies from the patients treated with TMZ (data not shown) were stained positively for p-OSR1. The patient with negative p-OSR1 expression did not receive TMZ treatment prior to the surgical removal of the tumor (data not shown). Future studies with increasing sample size of the recurrent GBMs with or without TMZ treatment are warranted and will allow us to validate whether TMZ treatment activates p-OSR1 in GBM.

In addition to WNK1 kinase, Haas et al. reported that WNK3 kinase is an important regulator of NKCC1 because of its elevated level in high-grade gliomas [6]. While robust expression of WNK1 kinase is also expressed in normal brain tissues and tumor tissues of all glioma grades [6]. Compared to normal human astrocytes, we detected a lower expression level of WNK3 protein in all three glioma cell lines (Additional file 1: Figure S4). The discrepancy of these findings on WNK1 and WNK3 expression may result from heterogeneity of the glioma cells.

Of note, GC#22 and GC#99 as well as U87 used in this study are O^6^-methylguanine-DNA methyltransferase (MGMT) negative (unpublished data). Therefore, it warrants further studies to validate our findings in MGMT-positive glioma cell lines.

### The WNK1/OSR1/NKCC1 signaling pathway in regulation of glioma cell migration

#### ***The WNK1/OSR1/NKCC1-mediated volume regulation and glioma cell migration***

NKCC1 activity is required in glioma cell migration [5-7]. In the current study, we found that GC#22 exhibited a slower basal random movement and transwell migration than GC#99, which is consistent with its low migration profile in the corresponding GSC xenografts [20]. We also documented that GC#22 and U87 migratory behaviors were significantly enhanced in the presence of TMZ. Either inhibition of NKCC1 with BMT or knockdown of WNK1 and OSR1 with siRNAs abolished the TMZ-mediated stimulation in GC#22 migration. On the other hand, there was no change in cell migration in GC#99 in response to TMZ. But, inhibition of NKCC1 by BMT significantly reduced basal levels of GC#99 mobility and transwell migration. Knockdown of WNK1 by siRNA also significantly reduced the basal migration of both GC#99 and GC#22. These findings suggest that the WNK1/OSR1/NKCC1 signaling pathway plays a role in GC migration either under basal conditions or in response to the TMZ-mediated stress. It has been reported that TMZ (50 μM) treatment enhanced U87 migration [21]. We speculate that the possible underlying mechanisms include stimulating the WNK/OSR1/NKCC1 cascade.

Precise regulation of the cell volume is an essential element for coordinated cell migration. A migrating cell has to actively govern cell volume regulatory ion transport mechanisms in order to achieve the appropriate morphological alteration [22]. NKCC1 protein could be involved in GC migration by regulating cell volume. Others and we have demonstrated that NKCC1 is the key regulator of cell volume in glioma cells [7,8]. Pharmacological inhibition of NKCC1 or genetically suppression of NKCC1 not only significantly abolishes active cell volume regulations in glioma cells [7,8], but also reduces glioma cell migration in transwell apparatus and in xenograft tumor tissues [5-7]. Moreover, we have found that TMZ treatment triggers activation of NKCC1 and in turn induces active cell volume regulatory in GCs [8]. We reported that inhibition of NKCC1 with its potent inhibitor BMT significantly impaired the replenishment of K^+^_i_, Cl^-^_i_ and attenuated RVI in GCs in the presence of TMZ. In the present study, we further discovered that NKCC1 and its regulatory kinases have an impact on volume regulation and glioma cell migration. Knockdown of the NKCC1 upstream kinase WNK1 by siRNA triggered significant loss of K^+^_i_ and Cl^-^_i_ and impaired the NKCC1-mediated RVI in GCs. These results strongly suggest that TMZ-mediated stimulation of the WNK1/OSR1/NKCC1 cascade has dual effects on glioma, it counteracts against loss of K^+^_i_, Cl^-^_i_ and AVD in order to promote GC survival, and it also functions to maintain focal cell volume regulation and facilitates glioma migration.

#### ***Phosphorylation and interactions of NKCC1 and ezrin in GC migration***

Cytoskeletal rearrangements and adhesion dynamics are indispensable prerequisites for cell migration [23]. The ERM proteins are closely related members of the band 4.1 superfamily of proteins [24]. Upon activation, ERM proteins act as linkers interacting with membrane proteins and the actin cytoskeleton. This specific function suggests ERM proteins are essential for many fundamental cellular processes, including determination of the cell shape, polarity, surface structure, cell adhesion and motility [24]. ERM proteins, especially ezrin, have an important role in cancer invasion and metastasis through regulation of adhesion molecules, participation in cell signal transduction, and signaling to other cell membrane channels in the tumor [25,26]. Garzon-Muvdi and colleagues demonstrated that in glioma cells, ezrin protein binds to the clusters of positively charged amino acids in carboxy-terminus domain of NKCC1, and mutation of the amino acids in these clusters reduced interaction between NKCC1 and ezrin [5].

In the current study, we concurrently detected an increase in ezrin protein pulled out from the same immunoprecipitation fractions, in which we observed increased expression of p-NKCC1 proteins in response to TMZ stress. Moreover, p-ERM expression was also significantly increased under these conditions. These findings suggest that phosphorylation of NKCC1 and ERM proteins may enhance their interactions. Several studies suggest that ERM proteins are phosphorylated and activated by the small GTPase Rho [27,28] or Akt kinase [28]. Interestingly, Akt has been shown to be activated by TMZ treatment in several types of cancer cells, such as pituitary adenoma [29], breast carcinoma [30], and metastatic melanoma [31]. Moreover, WNK1 has been suggested as a substrate of Akt kinase [9]. WNK1 is involved in regulation of Nogo-induced RhoA activation in PC12 neuronal cells. Knockdown of WNK1 by siRNA dramatically decreases the elevation of RhoA activity by Nogo treatment [32]. Therefore, it is possible that TMZ treatment may concurrently stimulate both phosphorylation of NKCC1 and ERM complex following activation of the WNK1 and Akt-mediated cascades, which enhances interactions between NKCC1 and ERM proteins during GC migration.

It is noteworthy that three different glioma cell lines responded differently to BMT treatment in our study. There is an apparent correlation between increased sensitivity to BMT treatment and higher basal cellular motility in these cell lines. GC#99, with aggressive profile and higher basal motility [20], demonstrated a more profound reduction in cell migration upon BMT treatment. However, when we compared whether p-NKCC1 and t-NKCC1 are differentially expressed among these cell lines, no significant differences were detected (Figure 1A and B). Studies have shown that an increase in translocation of NKCC1 protein from intracellular stores to the cytoplasmic membrane surface affects its activity in chondrocyte, parietal, and chief cells [33,34]. Thus, there might be differential NKCC1 expression on the cytoplasmic membrane in GC#99 versus other two cell lines, which may cause the difference of BMT sensitivities. Moreover, the expression level of t-WNK1 is significantly higher in GC#99, compared to GC#22 (127.1 ± 7.7%, p < 0.05) and U87 (173.9 ± 10.0%, p < 0.05). Whether the elevated kinase expression plays a role in NKCC1 protein trafficking, it warrants further investigation.

## Conclusion

**Figure 8:**
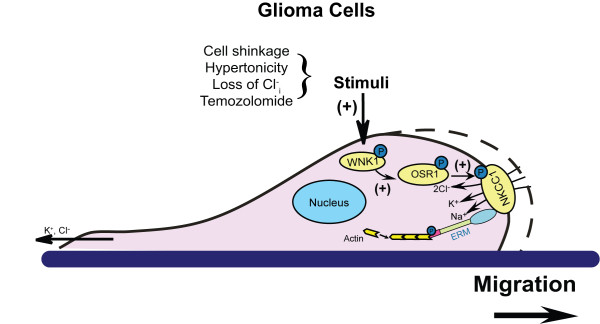
**Proposed mechanisms underlying TMZ-mediated regulation of GCs migration.** In GBM cells, WNK1 and OSR1 are the major kinases regulating NKCC1 activity under physiological condition and in the presence of TMZ treatment. WNK1/OSR1 kinases function in stimulation of NKCC1 activity to maintain K^+^_i_ and Cl^-^_i_ and cell volume homeostasis in the leading edge to promote cell migration. In addition, WNK1 may increase interactions between NKCC1 and ezrin to stimulate cell cytoskeletal rearrangements and further promote migration. Taken together, our findings suggest that targeting the WNK1/OSR1/NKCC1 pathway may synergistically reduce GBM cancer cell survival and migration in conjunction with TMZ treatment.

In summary as described in Figure 8, we concluded that WNK1 and OSR1 are the important upstream kinases in regulating NKCC1 activity in GBM cells in response to physiological (low Cl^-^_i_, cell shrinkage) or non-physiological stress (TMZ-mediated stress). WNK1/OSR1 kinases function in stimulation of NKCC1 activity to maintain intracellular K^+^ and Cl^-^ and cell volume homeostasis, which is important for glioma migration. In addition, phosphorylation of NKCC1 enhances its interactions with ezrin to stimulate cell cytoskeletal rearrangements and further promotes cell migration. Taken together, these novel findings suggest that combination of TMZ-mediated chemotherapy with inhibition of the WNK1/OSR1/NKCC1 signaling pathway presents a new strategy for improving glioma treatment.

## Materials and methods

### Materials

Gramicidin, nigericin, tributyltin, valinomcycin, TMZ, and BMT were purchased from Sigma Chemicals (St. Louis, MO). Dulbecco’s Modified Eagle Medium (DMEM), accutase, goat anti-IgG secondary antibodies Alexa Fluor^®^ 488 and 546, PBFI-AM, calcein-AM, MQAE, and pluronic acid were obtained from Invitrogen (Carlsbad, CA). Elite Vector Stain ABC System and 3-3′-diaminobenzidine (DAB) were from Vector Laboratories (Burlingame, CA). Rabbit anti-WNK1 (t-WNK1) (N-20, #sc-20468) was from Santa Cruz Biotechnology (Dallas, TX). Rabbit anti-phospho-WNK1 (p-WNK1) (T60, #AF4720) was from R&D Systems (Minneapolis, MN). Sheep anti-WNK3 (S156C) was developed previously described [35]. Mouse anti-NKCC (T4) antibody was from Developmental Studies Hybridoma Bank (Iowa City, IA). Mouse anti-α-tubulin (#2125), rabbit anti-ezrin (#3145), rabbit anti-ezrin/radixin/moesin (ERM) (#3142) and rabbit anti-phosphorylated-ERM (#3149) antibodies were from Cell Signaling (Beverly, MA). Rabbit anti-phosphorylated-NKCC1 (p-NKCC1) antibody (R5) was a kind gift from Dr. Biff Forbush (Yale University). Sheep anti-phosphorylated-NKCC1 recognizing the same residues (Thr^212^ and Thr ^217^) as R5 was developed as previously described [35]. Rabbit anti-SPAK/OSR1 (t-SPAK/t-OSR1) and rabbit anti-phosphorylated-SPAK (Ser^373^)/OSR1 (Ser^325^) (p-SPAK/p-OSR1) were developed as described before [36,37].

### Cell culture and GBM tissues

All studies involving human tissues were performed with approval from the University of Wisconsin-Madison and University of Pittsburgh Institutional Review Board with informed consent obtained from patients.

Primary glioma cell lines (GC#22 and GC#99) were established as described before [38]. U87 cell line was purchased from American Type Culture Collection. All glioma cell lines were grown in adherent cultures and maintained in DMEM supplemented with 10% FBS. Cultures were passaged approximately every 4 days with fresh medium at a density of 10^6^ cells/75 cm^2^ in a culture flask. Passages of 16–40 were used in this study.

Human cortex fetal neural stem cells (NSCs) and human astrocytes (a kind gift from Dr. Clive Svendsen) were used as a control and maintained as previously described [39].

### Immunofluorescence and immunohistochemistry

Immunofluorescence staining was conducted on xenografts of NOD-SCID mouse brains implanted with glioma as described before [38]. Briefly, 10 μm formalin-fixed, paraffin-embedded tissue sections were mounted on microscope slides. Tissue sections were deparaffinized and rehydrated to water, and microwaved in antigen unmasking solutions (Vector laboratories) for 20 min to retrieve epitopes. Sections were then incubated with a blocking solution (0.3% Triton X-100/5% goat serum) for 60 min at room temperature (RT) and with primary antibodies (sheep anti-p-NKCC1 1:100, mouse anti-t-NKCC1 1:100) overnight at 4°C. After rinsing in phosphate buffered saline (PBS) for 15 min, tissue sections were incubated with respective secondary antibodies conjugated to Alexa Fluor^®^ 488 or Alexa Fluor^®^ 546 (1:200 dilution) for 2 h at RT. Sections were then rinsed and incubated with To-pro-3 iodide (1:1000 in antibody diluting solution) for 15 min at RT and mounted with Vectashield mounting medium. Fluorescence images were captured with a Leica DMIRE2 inverted confocal laser-scanning microscope under the 40× oil immersion objective lens. Samples were excited at 488 nm (argon/krypton), 543 nm, and 633 nm. The emission fluorescence was recorded at 512–548 nm, 585–650 nm, and 650–750 nm, respectively.

For immunohistochemistry study, sections were blocked for endogenous peroxidase and biotin before the application of the primary antibody. Incubation of primary antibodies (rabbit-anti-p-SPAK/OSR1 1:50, mouse anti-t-OSR1 1:50) was conducted overnight at 4°C. Incubation of secondary antibodies was applied for 2 h at RT. Subsequent immunodetection was conducted using the Elite Vector Stain ABC System. Color visualization was conducted using DAB as the chromagen substrate. Tissues were counterstained with hematoxylin to visualize cellular morphology. Images were acquired with a Nikon TE-2000 brightfield microscope.

### GBM tissue microarray

A tissue microarray from 205 GBM patients diagnosed between 1999 and 2009 was created from the UW Department of Pathology and Laboratory Medicine archives as described [20]. Out of 205 patients, 138 patients had a recorded value for overall survival and a preserved tissue punch. Diagnosis and tissue punch location were defined by neuropathology prior to incorporation into microarray [20]. Rabbit anti-p-SPAK/OSR1 (1:50) was used to label the tissue microarray. Each punch was subjectively scored for negative (score 0), mild (score 1), moderate (score 2) and strong (score 3) of p-SPAK/OSR1 expression by light microscopic visualization of intensity of cytoplasmic DAB. Nuclear or fibrillary labeling was not scored as positive. In cases of multiple punches/cores for one patient tumor sample, the score given represented the most frequent expression level.

### Cell motility measurement with time-lapse imaging

Glioma cells on 35 mm cell culture dishes were placed in a stage top incubator at 37°C with 5% CO_2_ + 95% air (TIZ model, Tokai Hit; Shizuoka-ken, Japan). Motility of cells was monitored under 10× objective lens with a time-lapse video microscope system (the Nikon TiE 300 inverted epifluorescence microscope) and MetaMorph software (Molecular Devices; Sunnyvale, CA, USA). Time-lapse DIC images were acquired in 5-min intervals for 5 h either under control conditions (DMEM supplemented with 10% FBS) or in the presence of 10 μM BMT, 100 μM TMZ, or 100 μM TMZ plus 10 μM BMT. Images were analyzed by ImageJ software (National Institute of Health, USA) and cell tracking was performed using the Manual Tracking plugin. Total distance traveled was determined by tracking the movement of the cell gravity center, and its coordinates were used to calculate the distances. The slope of the curve was obtained as averaged speed.

### Serum-induced microchemotaxis assay

Transwell membrane cell culture inserts (8.0 μm pore size, Becton Dickinson) were coated with 0.5 μg/ml poly-d-lysine overnight at RT and washed in PBS for 5 min for 3 times. Dissociated GCs (4 × 10^4^ cells) in 100 μl serum-free DMEM with different treatment regimens (control medium, 10 μM BMT, 100 μM TMZ, or 100 μM TMZ plus 10 μM BMT) were seeded on the top of the membrane insert, and the lower wells contained 700 μL DMEM plus 10% FBS. After incubation for 5 h in the cell culture incubator at 37°C, cells were fixed with 4% paraformaldehyde (PFA) and non-migrated cells on the inserts were wiped off with cotton q-tips. The migrated cells on the bottom surface were subjected to DAPI staining (2 μg/mL in PBS) for 15 min at RT. The membranes were removed and inertly mounted on microscope slides. Slides were excited at 358 nm with a xenon lamp and the emission fluorescence at 461 nm recorded with a Princeton Instruments MicroMax CCD camera attached to the Nikon TiE microscope using the MetaMorph software. Images of 5 random fields were captured under the 40× objective lens. Migrated cells in all 5 fields were averaged to give a mean cell count for each experiment.

### Intracellular Cl^-^ concentration ([Cl^-^]_i_) measurement

The fluorescent dye MQAE was used to determine [Cl^-^]_i_ as described by Rocha-Gonzalez [40] with some modifications [8]. Cells were incubated with 5 mM MQAE for 1–2 h (37°C) in a HEPES buffered isotonic solution. The HEPES buffered isotonic solution contained (in mM, pH 7.4): 100 NaCl, 5.4 KCl, 1.3 CaCl_2_, 0.8 MgSO_4_, 20 HEPES, 5.5 glucose, 0.4 NaHC0_3_, and 70 sucrose with 310 mOsm determined with an osmometer (Advanced Instruments, Norwood, MA). The coverslip was placed in the heated imaging chamber for 30 min before imaging. Using the Nikon TiE inverted epifluorescence microscope and the 40× oil immersion objective lens, cells were excited every 60 sec at 340 and emission fluorescence at 460 nm recorded. Images were collected and analyzed with the MetaFluor image-processing software. At the end of each experiment, the MQAE florescence was calibrated under a steady state condition when [Cl^-^]_o_ and [Cl^-^]_i_ were considered equal by exposing cells to a series of calibration solutions containing 10 μM tributylin and 5 μM nigericin (Rocha-Gonzalez, Mao, and Alvarez-Leefmans 169–84). The series of Cl^-^ calibration solutions contained (in mM): 1.27 Ca(OH)_2_, 0.8 MgSO_4_, 5 HEPES, 5.5 glucose, 120 K^+^, and variable Cl^-^ and NO_3_^-^. In these solutions, Cl^-^ was varied from 0 to 60 mM keeping the sum of Cl^-^ and NO_3_^-^ equal to 120 mM. KSCN (150 mM) was used to quench the MQAE fluorescence, which was taken as background fluorescence. [Cl^-^]_i_ was determined from the MQAE fluorescence (drift-corrected, background-corrected) using the following equation: [*Cl*^-^]_*i*_ = [(*F*_*o*_/*F*_*t*_) - 1)]/*Ksv*, where F_o_ was the fluorescence in 0 mM [Cl^-^]_o_, Ft was the fluorescence at any given time point, and K_sv_ was the slope of the linear fit of MQAE fluorescence vs. the [Cl^-^]_o_ of the standards. A K_sv_ of 13.4 ± 1.5 M^-1^ was calculated in our study [8], a value similar to that reported by others [40].

### Intracellular K^+^ concentration ([K^+^]_i_) measurement

[K^+^]_i_ was determined by a modified method as described by Kiedrowski [41]. Briefly, cells were incubated with 5 μM PBFI-AM plus 0.02% pluronic acid at 37°C for 90 min. The coverslips were placed in the heated imaging chamber at 37°C. Cells were rinsed and images collected using the Nikon TiE inverted epifluorescence microscope equipped with the 40× oil immersion objective lens. Cells were excited every 20 sec at 340 and 380 nm and the emission fluorescence was recorded at 510 nm. Images were analyzed with the MetaFluor image-processing software. At the end of each experiment, a calibration was performed by exposing cells to standards of varying K^+^ concentrations plus gramicidin and valinomcycin (10 μM each). K^+^ standards contained 30 mM NaCl, 20 mM HEPES, and 1 mM MgCl_2_, with varied K^+^ from 20 to 120 mM by substituting K^+^-gluconate and LiCl such that the sum of K^+^ and Li^+^ was 100 mM.

### RNA interference knockdown of WNK1 or OSR1

Knockdown of WNK1 or OSR1 protein expression was induced by small interfering RNA (siRNA). The scramble siRNA (Silencer^®^ Negative Control No. 1 siRNA, Cat. No. AM4635) and siRNAs targeting human WNK1 (ID: s35235) and OSR1 (ID: s19302) were purchased from Invitrogen. The sequences of WNK1 siRNAs are: sense 5′-CAAUGSGUCAGAUAUACGAAtt-3′; antisense 5′-UUCGAUAUCUGACUCAUUGtc-3′; OSR1 siRNA: sense 5′-GAACCUCAGUCAAAUCGAUtt-3′; antisense 5′-AUCGAUUUGACUGAGGUUCtt-3′. Dissociated GCs were seeded in 6-well plates (10^5^ cells/well/2 ml) in DMEM plus 10% FBS at 24 h prior to transfection. Lipofectamine RNAiMAX/siRNA complexes were prepared by adding siRNA (15 nM as a final concentration) and 5 μl of Lipofectamine RANiMAX (Invitrogen, Carlsbad, CA) in 500 μL serum-free optiMEM. Complexes were allowed to form at RT for 10 min and added to each well in the 6-well plate. The cells were incubated at 37°C and subjected to experiments 48 h after transfection.

### Immunoblotting assay

Cells were washed with ice-cold PBS that contained 2 mM EDTA and protease inhibitors as described before [42]. Cells were lysed by sonication on ice. Protein content of the cellular lysate was determined with BCA Protein Assay Kit (Pierce, Rockford, IL). Samples (30 μg lysate protein) were denatured in SDS reducing buffer (1:2 vol/vol) and heated at 90°C for 5 min, and then electrophoretically separated on 8-10% SDS gels. After transferring to PVDF membranes, the blots were blocked in 7.5% nonfat dry milk with Tris buffered saline for 1 h at RT and incubated with a primary antibody at 4°C overnight. The blots were probed with monoclonal T4 antibody against total NKCC1 (1:2000), polyclonal antibody (R5) against a diphosphopeptide containing T184 and T189 of shark NKCC1 (*p*-NKCC; 1:1000), rabbit anti-t-WNK1 (1:500), rabbit anti-p-WNK1 (1:1000), rabbit anti t-SPAK/OSR1 (1:200) or rabbit anti-p-SPAK/OSR1. After rinsing, the blots were incubated with horseradish peroxidase-conjugated secondary IgG for 1 h at RT. Bound antibody was visualized with an enhanced chemiluminescence assay (Amersham, Piscataway, NJ).

Immunoprecipitation was conducted to examine interactions between NKCC1 and ezrin proteins using the Pierce^®^ Classic IP Kit (ThermoScientific, Rockford, IL, USA). Cellular lysate samples (0.2 mg protein) were incubated with 2 mg of mouse anti-t-NKCC1 antibody at 4°C overnight. Immunocomplexes were mixed with 20 μL protein A/G beads (50% slurry) in a Pierce spin column and incubated for 2 h. The immunocomplexes were washed and dissociated from beads with the Laemmli sample buffer and heated at 95°C for 10 min. The resolved proteins and prestained molecular mass markers were separated on 7.5% SDS-PAGE. The blots were probed for t-ezrin, p-NKCC1, and t-NKCC1 (for equal total protein input control). Densitometric measurement of each protein band was performed using the Gel Analysis Tool in Image J.

### Statistical analysis

The results are expressed as the mean ± SEM. Comparisons between groups were made by Student’s t-test or one-way ANOVA using the Bonferroni post-hoc test in the case of multiple comparisons (SigmaStat, Systat Software, Point Richmond, CA, USA). p < 0.05 was considered statistically significant. n values represent the number of independent cultures or tissue samples.

## Abbreviations

AVD: Apoptotic volume decrease; BMT: Bumetanide; ERM: Ezrin, radixin, and moesin; GBM: Glioblastoma multiforme; MGMT: O^6^-methylguanine-DNA methyltransferase; NKCC1: Na^+^-K^+^-2Cl^-^ cotransporter isoform 1; OSR1: Oxidative stress-responsive kinase-1; SPAK: SPS1-related proline/alanine-rich kinase; RVI: Regulatory volume increase; TMZ: Temozolomide; WNK1: With-No-K (Lysine) kinase 1.

## Competing interests

The authors declare that they have no competing interests.

## Authors’ contributions

Conceived and designed the experiments: DS, SSY, SHL, KK, JSK. Performed the experiments: WZ, GB. Analyzed the data: WZ, KP, PC. Wrote the manuscript: WZ, GB, DS, KK, JSK. All authors read and approved the final manuscript.

## Supplementary Material

Additional file 1: Figure S1 Visualization of expression of SPAK in GCs after extended ECL exposure. **Figure S2.** BMT abolished TMZ-stimulated cell migration in U87. **Figure S3.** Total protein expression of WNK1/SPAK/OSR1/NKCC1 signaling pathway does not change in the presence of TMZ. **Figure S4.** Low expression of WNK3 in glioma cell lines. Click here for file
