# Supplementary material for: WNK1-OSR1 kinase-mediated phospho-activation of Na+-K+-2Cl- cotransporter facilitates glioma migration
Source: Mol Cancer. 2014 Feb 20;13:31. doi: 10.1186/1476-4598-13-31 (PMC3936893; doi:10.1186/1476-4598-13-31)
Supplement: Additional file 1: Figure S1 — Visualization of expression of SPAK in GCs after extended ECL exposure. Figure S2. BMT abolished TMZ-stimulated cell migration in U87. Figure S3. Total protein expression of WNK1/SPAK/OSR1/NKCC1 signaling pathway does not change in the presence of TMZ. Figure S4. Low expression of WNK3 in glioma cell lines. [file 1476-4598-13-31-S1.doc]

**Additional file 1**

**Figure S1. Visualization of expression of SPAK in GCs after extended ECL exposure**

**
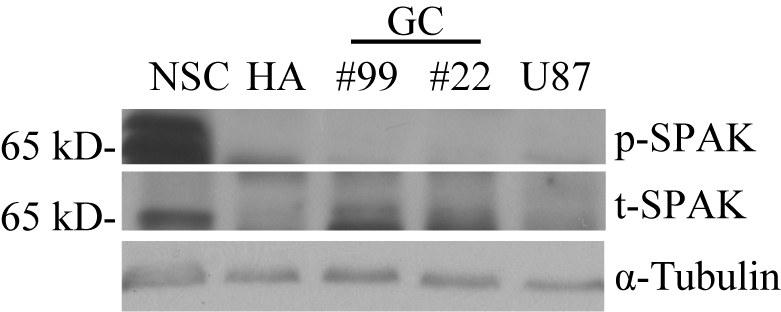
**

Representative immunoblots for detection of either phosphorylated (p-) or total (t-) SPAK expression with extended ECL exposure (3 h), which further indicates the relative low expression of SPAK protein in glioma cells (GC) and human astrocytes (HA), compared to human neural stem cells (NSC).

**Figure S2. BMT abolished TMZ-stimulated cell migration in U87**

**
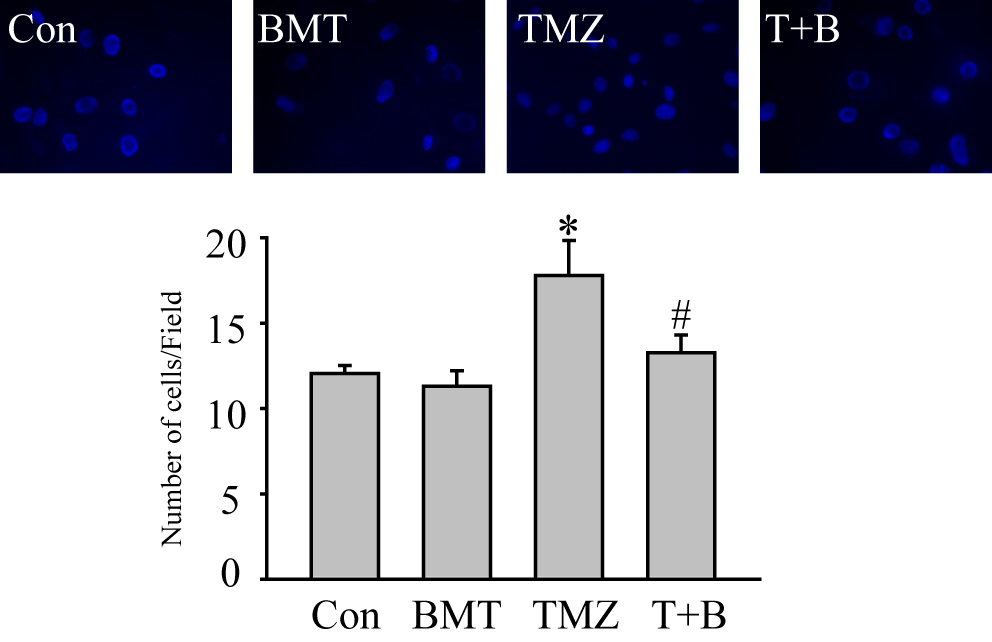
**

Upper panel: representative images of U87 cells that have migrated through an 8-µm transwell barrier after 5 h were shown. Cells (4 × 10^4^ cells) in 100 μl serum-free DMEM with different treatment regimens (control medium (Con), 10 µM bumetanide (BMT), 100 µM TMZ, or 100 µM TMZ plus 10 µM BMT (T+B)) were seeded on the top of the membrane insert and allowed for migration for 5h. Lower panel: Summary data of numbers of migrated cells in different treatment groups. Data are mean ± SEM. n = 4, *p < 0.05 vs. Con. #p<0.05 vs. TMZ.

**Figure S3. Total protein expression of WNK1/SPAK/OSR1/NKCC1 signaling pathway does not change in the presence of TMZ**

**
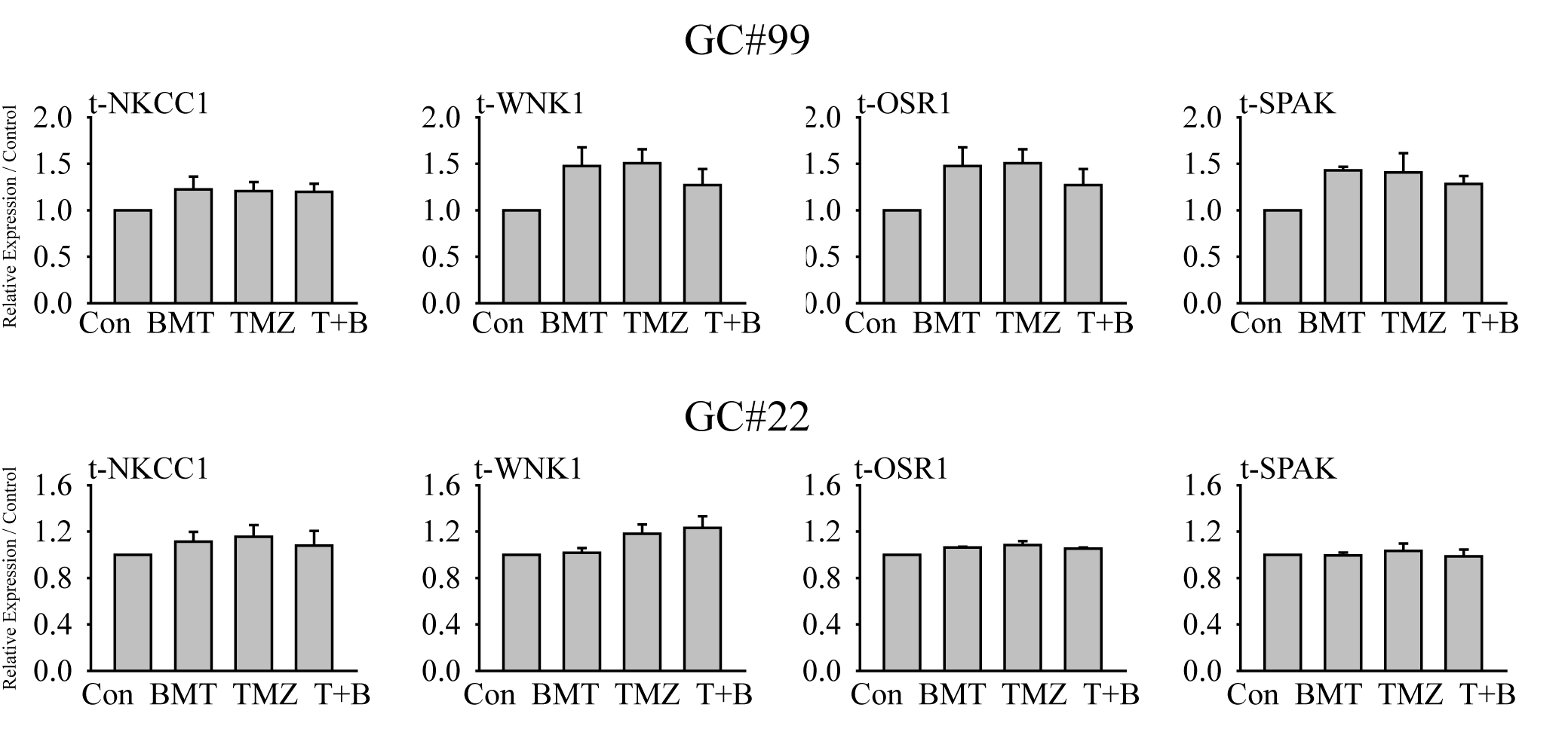
**

Summary data of immunoblotting. GCs were exposed to control medium (Con), 10 µM bumetanide (BMT), 100 µM TMZ, or 100 µM TMZ plus 10 µM BMT (T+B) for 4h. Expression of each protein was first normalized by α-tubulin. Relative expression level in different treatments was then normalized to Con. Data are mean ± SEM. n = 4-5.

**Figure S4. Low expression of WNK3 in glioma cell lines**

**
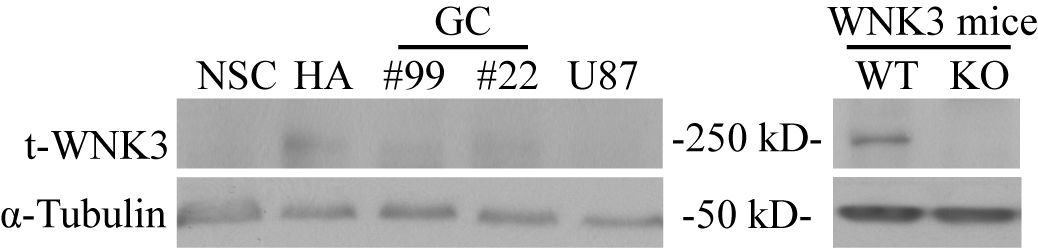
**

Left panel: Representative immunoblots for expression of total (t-) WNK3 in different cell lines, which indicates low expression of t-WNK3 in glioma cells, human astrocytes (HA), and human neural stem cells (NSC). Right panel: Brain tissues from WNK3 wild-type (WT) and targeted WNK3 knockout mice (KO) were used to validate the presence and absence of WNK3 protein expression. Cell lysate of cultures or brain tissue homogenate (30 µg protein) was used in this experiment. More, characterizations of WT and KO WNK3 mice are conducted in another study in our laboratory (data not shown).
